# Kompetenzentwicklung in der Medizininformatik-Initiative (MII) – Lehrangebote für einen souveränen und sicheren Umgang mit medizinischen Daten

**DOI:** 10.1007/s00103-024-03881-x

**Published:** 2024-05-15

**Authors:** Petra Knaup-Gregori, Martin Boeker, Toralf Kirsten, Dagmar Krefting, Erik Schiller, Paul Schmücker, Christina Schüttler, Anne Seim, Cord Spreckelsen, Alfred Winter

**Affiliations:** 1 Institut für Medizinische Informatik, Universitätsklinikum Heidelberg, Im Neuenheimer Feld 130.3, 69120 Heidelberg, Deutschland; 2 Institut für Künstliche Intelligenz und Informatik in der Medizin, Klinikum rechts der Isar, School of Medicine and Health, Technische Universität München, München, Deutschland; 3 Abteilung für Medical Data Science, Medizininformatikzentrum, Universitätsklinikum Leipzig, Leipzig, Deutschland; 4 Institut für Medizinische Informatik, Universitätsmedizin Göttingen, Göttingen, Deutschland; 5 MFT Medizinischer Fakultätentag der Bundesrepublik Deutschland e. V., Berlin, Deutschland; 6 Institut für Medizinische Informatik, Hochschule Mannheim, Mannheim, Deutschland; 7 Medizinisches Zentrum für Informations- und Kommunikationstechnik, Universitätsklinikum Erlangen, Erlangen, Deutschland; 8 Institut für Medizinische Informatik und Biometrie, Technische Universität Dresden, Dresden, Deutschland; 9 Institut für Medizinische Statistik, Informatik und Datenwissenschaften, Universitätsklinikum Jena, Jena, Deutschland; 10 Institut für Medizinische Informatik, Statistik und Epidemiologie, Universität Leipzig, Leipzig, Deutschland

**Keywords:** Digitale Kompetenzen, Digitale Medizin, E‑Learning, Medizinische Datenwissenschaften, Medizinische Informatik, Digital literacy, Digital medicine, eLearning, Medical data science, Medical informatics

## Abstract

Um die Ziele der Medizininformatik-Initiative (MII) erreichen zu können, wird Personal mit Kompetenzen im Bereich der medizinischen Informatik und Datenwissenschaften benötigt. Um diese Kompetenzen zu fördern, wurden in jedem Konsortium Ausbildungsaktivitäten etabliert. Zudem wurden konsortiumübergreifende Aktivitäten etabliert. Dieser Artikel berichtet über die Konzepte, umgesetzte Programme und Erfahrungen. Es sind 51 neue Professuren und 10 neue Studienangebote eingerichtet worden: 1 Bachelor-, 6 konsekutive und 3 berufsbegleitende Masterstudiengänge. Die entwickelten Lern- und Fortbildungsangebote stehen standortübergreifend zur Verfügung. Darüber hinaus wurden Zertifizierungs- und Anerkennungsmöglichkeiten geschaffen.

Die Lehrangebote richten sich an Zielgruppen mit fachlichem Hintergrund in Informatik, Medizin, Pflege, Bioinformatik, Biologie, Natur- und Datenwissenschaften. Zusatzqualifikationen für Ärzt*innen in Informatik und für Informatiker*innen in Medizin erweisen sich als besonders wichtig. Sie können zu einer höheren Qualität bei der Softwareentwicklung und einer besseren Unterstützung der Behandlungsprozesse durch Anwendungssysteme beitragen.

In allen Konsortien waren digitale Lernmethoden wichtig. Sie bieten Flexibilität, um standortübergreifend und interprofessionell auszubilden. So können ein Lernen in individuellem Tempo und ein Austausch unter Berufsgruppen erreicht werden. Der Erfolg der MII hängt wesentlich von der gesellschaftlichen Akzeptanz der Mehrfachnutzung medizinischer Daten in Versorgung und Forschung ab. Die dafür erforderlichen Informationen werden durch die Öffentlichkeitsarbeit der MII bereitgestellt. In der Gesellschaft besteht ein enormer Bedarf an Kompetenz im Umgang mit medizinischen Daten und digitalen Werkzeugen.

## Abstract (en)

In order to achieve the goals of the Medical Informatics Initiative (MII), staff with skills in the field of medical informatics and data science are required. Each consortium has established training activities. Further, cross-consortium activities have emerged. This article describes the concepts, implemented programs, and experiences in the consortia. Fifty-one new professorships have been established and 10 new study programs have been created: 1 bachelorʼs degree and 6 consecutive and 3 part-time masterʼs degree programs. Further, learning and training opportunities can be used by all MII partners. Certification and recognition opportunities have been created.

The educational offers are aimed at target groups with a background in computer science, medicine, nursing, bioinformatics, biology, natural science, and data science. Additional qualifications for physicians in computer science and computer scientists in medicine seem to be particularly important. They can lead to higher quality in software development and better support for treatment processes by application systems.

Digital learning methods were important in all consortia. They offer flexibility for cross-location and interprofessional training. This enables learning at an individual pace and an exchange between professional groups.

The success of the MII depends largely on society’s acceptance of the multiple use of medical data in both healthcare and research. The information required for this is provided by the MII’s public relations work. There is also an enormous need in society for medical and digital literacy.

## Einleitung

Die im Jahr 2016 gegründete Medizininformatik-Initiative (MII) war von Beginn an auf einen längeren Zeitraum und die Schaffung einer nachhaltigen Infrastruktur für gemeinsame Datennutzung ausgelegt [1]. Im Rahmen der MII sollten Datenintegrationszentren (DIZ), Medizininformatik-Professuren und Nachwuchsgruppen eingerichtet und zunächst alle Universitätsklinika sowie nachfolgend weitere deutsche Krankenhäuser integriert werden.

Die medizinische Informatik trägt mit ihren Methoden und Werkzeugen dazu bei, dass Erkenntnisse aus medizinischen Daten gewonnen werden können (Datenwissenschaften). Um die angestrebten Ziele der MII erreichen zu können, besteht ein erheblicher Bedarf an Personal mit Kompetenzen in Medizininformatik und Datenwissenschaften. Bereits vor Beginn der MII war ein Fachkräftemangel auf diesem Gebiet spürbar [2]. Daher hat jedes Konsortium der MII eigene Ausbildungsaktivitäten etabliert. Zusätzlich sind konsortiumübergreifende Aktivitäten entstanden, sodass eine strategische Arbeitsgruppe (Taskforce) unter Mitwirkung des Medizinischen Fakultätentages (MFT) eingerichtet wurde. Nähere Informationen zur Struktur der MII mit ihren wissenschaftlichen Konsortien sind im Beitrag von Semler et al. in diesem Themenheft zu finden.

Dieser Artikel gibt eine Übersicht über die Konzepte, umgesetzten Programme und Erfahrungen in den einzelnen Konsortien. Bei den vielfältigen Aktivitäten handelt es sich um Aus‑, Weiter- und Fortbildungsangebote für Forschende und Anwendende in den Bereichen Medizin, Informatik, medizinische Informatik, medizinische Datenwissenschaften, Management und Sozialkompetenz. Dazu gehören Schulungs- und Sensibilisierungsangebote für Personen, die neu in Themen der MII arbeiten, wie ärztliche und pflegerische Fachkräfte sowie Mitarbeitende in Studien. Ergebnisse und weitere Entwicklung werden auch unter dem Blickwinkel der konsortiumübergreifenden Aktivitäten und weitergehenden Vernetzung reflektiert.

## Aktivitäten zur Kompetenzentwicklung der Konsortien der MII

Die spezifischen Aktivitäten zu Aus‑, Fort- und Weiterbildung werden zunächst in alphabetischer Reihenfolge der Konsortien vorgestellt. Anschließend berichten wir über Nachwuchsgruppen und konsortiumübergreifende Koordination. Mehrere Konsortien haben Spring, Summer, Autumn und Winter Schools angeboten. Diese werden im Folgenden als Saisonschulen bezeichnet.

### DIFUTURE

Das DIFUTURE-Konsortium umfasst Universitäten und Universitätsklinika an den Standorten Augsburg, Homburg, München: Ludwig-Maximilians-Universität (LMU) und Technische Universität (TUM), Regensburg, Tübingen und Ulm [3].

#### Konzept zur Kompetenzentwicklung.

DIFUTURE hat durch die Einrichtung neuer Professuren in den Bereichen Medical Data Sciences, Bioinformatik und Medizininformatik einen Schwerpunkt auf die Förderung des akademischen Nachwuchses in diesen Bereichen gelegt. Zusätzlich wurden Nachwuchsgruppen eingerichtet, um talentierten Forschenden eine Plattform zu bieten, ihre Karriere in der Medizininformatik voranzutreiben. Die Fortbildung von Mitarbeitenden wurde v. a. durch regelmäßige Retreats unterstützt, die ermöglichten, sich intensiv mit relevanten Themen auseinanderzusetzen, Wissen zu erweitern und sich über aktuelle Entwicklungen in der Medizininformatik auszutauschen. Um spezifische Inhalte zu vermitteln, wurden elektronische Lehrmaterialien erstellt, die innerhalb des Konsortiums genutzt werden können. Seit 2022 arbeitet DIFUTURE eng mit dem MIRACUM-Konsortium zusammen. Dies ermöglicht es, die Ressourcen zu bündeln und von den jeweiligen Stärken zu profitieren. Saisonschulen werden gemeinsam mit Lehrenden beider Konsortien durchgeführt. Im gemeinsamen MIRACUM-DIFUTURE-Kolloquium werden aktuelle Forschungsergebnisse und Entwicklungen präsentiert. Die Inhalte dieses Kolloquiums stehen elektronisch bereit.

#### Erfahrungen.

Folgende Professuren wurden eingerichtet und besetzt: 8 in Augsburg, 1 an der LMU, 4 an der TUM, 2 in Regensburg. Darüber hinaus hat DIFUTURE Bachelor- und Masterstudiengänge an den Standorten eingeführt. Zu den angebotenen Studiengängen gehören „Biomedical Computing“, „Medical Information Sciences“ und „Medical Informatics“. Diese Studiengänge ermöglichen es, fundierte Kenntnisse und Fähigkeiten in medizinischer Informatik, Bioinformatik und Data Science zu erwerben. Eine detaillierte Übersicht über die eingerichteten Professuren und angebotenen Lehrveranstaltungen findet sich auf der Website des Konsortiums^1^.

#### Ausblick.

Im Hinblick auf die zukünftige Kompetenzentwicklung in DIFUTURE steht der Auf- und Ausbau von interdisziplinären Lehr- und Studienangeboten im Vordergrund. Besonderes Augenmerk wird auf Nutzungsaspekte der MII und DIZ durch Wissenschaftler*innen und klinisches Personal gelegt, um Studierenden das notwendige Wissen und Verständnis für die effektive Nutzung von Daten der DIZ zu vermitteln. Ein weiterer Schwerpunkt sind die schnelle Einarbeitung und Weiterbildung der Mitarbeitenden in den DIZ.

### HiGHmed

Das HiGHmed-Konsortium umfasst aktuell 10 Universitätsklinika, ein außeruniversitäres Klinikum und eine außeruniversitäre Forschungseinrichtung [4].

#### Konzept zur Kompetenzentwicklung.

Die HiGHmed-Gründungsstandorte Heidelberg, Göttingen und Hannover sind Universitäten mit einer langen Tradition in der akademischen Ausbildung in medizinischer Informatik. Für die Kompetenzentwicklung in HiGHmed wurden von Beginn an etablierte Studiengänge im Bereich medizinische Informatik hinzugezogen, die ansonsten nicht an der MII beteiligt sind. Dies waren in Braunschweig die Technische Universität und das Helmholtz-Zentrum für Infektionsforschung sowie die Hochschulen Göttingen, Hannover und Heilbronn.

Durch jeden Standort wurden digitale Lernmodule für in der MII relevante Ausbildungsinhalte gemäß dem didaktischen Konzept der „e-tivities“ von Gilly Salmon [5] im Umfang von 6 Leistungspunkten (European Credit Transfer System – ECTS) mit ca. 6×30 h Arbeitsaufwand für Teilnehmende entwickelt. E‑tivities sind moderierte elektronische Aktivitäten auf Lernplattformen, die zur Interaktion anregen. Die Lernmodule sind in die lokalen Studiengänge integriert und offen für Teilnehmende von anderen Standorten. Die Lehrthemen ergänzen sich gegenseitig und reichen von „Assistierenden Gesundheitstechnologien“ über „Fortgeschrittene Konzepte der Datenanalyse“ bis hin zur „Sicheren Softwareentwicklung“. Für Teilnehmende ohne Einschreibung in die lokalen Studiengänge wurden 3 Zertifikate mit jeweils 18 ECTS entwickelt.

Neben dem einheitlichen didaktischen Konzept wurde ein Evaluationskonzept für die digitalen Lernmodule entwickelt und standortübergreifend genutzt [6, 7].

#### Erfahrungen.

Es wurden 11 Lernmodule wiederholt mit insgesamt ca. 760 Teilnehmenden durchgeführt [8–10]. Da die MII bereits vor der COVID-19-Pandemie startete, waren die beteiligten Studiengänge gut gerüstet für Online-Lehre, die deutlich über Vorlesungen als Webkonferenz oder Vorlesungsaufzeichnungen hinausgeht [11].

Die beteiligten Studiengänge haben die Lernmodule von anderen HiGHmed-Standorten anerkannt. Dies muss häufig individuell gelöst werden, weil in den Bundesländern und Prüfungsordnungen unterschiedliche Regelungen bestehen. Der administrative Aufwand ist teilweise hoch.

Aus HiGHmed ist der Podcast „Digitalisierung der Medizin“ hervorgegangen.^2^ Es werden seit 2019 aktuelle Themen der medizinischen Informatik von bisher über 40 Frauen anhand ihrer Karrierewege vorgestellt und inhaltlich diskutiert. Bis Ende 2023 sind 24 Folgen erschienen mit über 32.000 Downloads.

#### Ausblick.

Die Evaluationen haben gezeigt, dass sich das didaktische Konzept und die Modularität für die digitalen Lerneinheiten bewährt haben. Die Lehrenden haben die Erfahrung gemacht, dass das Konzept – insbesondere bei unterschiedlichen Lernvoraussetzungen – hohe Flexibilität bietet. Die erarbeiteten Konzepte und Lernmodule werden an den einzelnen Standorten weiter genutzt. Allerdings zeigt sich in der grundständigen Ausbildung ein starker Wunsch nach Präsenzveranstaltungen. Dies erschwert eine standortübergreifende Teilnahme an vollständigen Kursen über ein ganzes Semester. Daher werden die Lerninhalte als digitale Microlearning-Einheiten weiterentwickelt, die in unterschiedlichen Lerneinheiten und Studiengängen über eine gemeinsamen E‑Learning-Plattform genutzt werden können. Diese ist an die Authentifizierungs- und Autorisierungsinfrastruktur des Deutschen Forschungsnetzes e. V. (DFN-AAI) angebunden, sodass Beteiligte sich per Einmalanmeldung („single-sign-on“) anmelden können.

Die Kooperation mit dem durch das Land Niedersachsen geförderten Zukunftslabor Gesundheit (ZLG) führte zu weiteren Lernmodulen nach dem didaktischen Konzept der E‑tivities [4]. Um hierfür die Zielgruppe der Bürger*innen erreichen zu können, wurden Lernziele in den Bereichen Basiswissen und Werte ergänzt mit Themen, wie z. B. bereits eingesetzte digitale Technologien oder das Für und Wider des Datenspendens.

### MIRACUM

Zum MIRACUM-Konsortium gehören 11 Standorte: Erlangen, Dresden, Greifswald, Magdeburg, Marburg, Gießen, Mannheim, Frankfurt, Mainz, Freiburg und Chemnitz [12]. Seit Sommer 2022 wird eng mit dem DIFUTURE-Konsortium kooperiert, nicht nur in Aus‑, Weiter- und Fortbildung [13].

#### Konzept zur Kompetenzentwicklung.

Zum Abbau des IT-Fachkräftemangels, zur lebenslangen Weiterbildung der IT-Fachkräfte und zur grundständigen Qualifizierung hat MIRACUM neben dem berufsbegleitenden onlinebasierten Masterstudiengang „Biomedizinische Informatik und Data Science“ (BIDS) weiterbildende Zertifikatskurse und -programme, wöchentliche Kolloquien für die Fach-Community sowie Fortbildungsprogramme und Hospitationen eingerichtet.

Der akkreditierte BIDS-Studiengang spricht Absolvent*innen aus den Bereichen Informatik, Medizin, Natur- und Biowissenschaften an. Schwerpunkte des Studiums sind Medizin, Informatik, medizinische Informatik, Biomedical Data Science sowie Management & Social Skills [14, 15]. Der Studiengang startete im Oktober 2020 mit 27 Lernmodulen. Er wurde von der Hochschule Mannheim mit Unterstützung von MIRACUM und der Graduate School Rhein Neckar gGmbH eingerichtet. Im Rahmen von Projekt- und Masterarbeiten werden Fragestellungen aus den MIRACUM- und DIFUTURE-Standorten bearbeitet.

Ständige Weiter- und Fortbildung ist für alle in der MII Tätigen unabdingbar. Daher bieten MIRACUM und DIFUTURE regelmäßig onlinebasierte zertifizierte Weiterbildungskurse an. Bis Ende 2023 haben 62 Personen an dem Zertifikatsprogramm teilgenommen und 157 Zertifikate erworben.

Das *Online-Kolloquium* von MIRACUM und DIFUTURE informiert die Community in ca. 30 min, inkl. Diskussion, über neue Entwicklungen. Das Kolloquium startete 2017. Bis Ende 2023 wurden 311 Kolloquien jeweils mit 40 bis 100 Teilnehmenden durchgeführt. Ein digitales Archiv mit den Foliensätzen und Videoaufzeichnungen der letzten 71 Kolloquien wurde aufgebaut.^3^

Das *Hospitationsprogramm* spricht primär Mitarbeitende der DIZ und der Use Cases an. Expert*innen aus den Kompetenzzentren von MIRACUM und DIFUTURE laden zu sich ein und stellen relevante Themen vor, wie z. B. Aufsetzen und Nutzen der Softwarelösung ProSkive, das Kodiersystem LOINC als Bestandteil eines erweiterten „Medical Data Repository“ sowie die europäische Medizinprodukte-Verordnung (Medical Device Regulation). Es wird über Erfahrungen bei Entwicklung, Einführung und Betrieb berichtet. Bisher wurden 32 Hospitationen für ca. 380 MIRACUM- und DIFUTURE-Mitarbeitende durchgeführt.

Seit 2018 fanden 8 Fortbildungsveranstaltungen in Form von 5‑tägigen Saisonschulen an verschiedenen Orten statt. Sie hatten Leitthemen wie „Werkzeuge der MII“ und „Alte und Neue Use Cases der MII“.

#### Erfahrungen und Ausblick.

MIRACUM wird sich auch in den kommenden Jahren der lebenslangen Fortbildung von im Gesundheitswesen tätigen Menschen mit zielgruppenspezifischen Angeboten widmen. Insbesondere die Hospitationen und Saisonschulen mit ihren interaktiven Praxisanteilen haben sich als probate Mittel erwiesen und werden fortgeführt.

### SMITH

Das SMITH-Konsortium umfasst 9 Universitätsklinika, 4 Universitäten, 2 außeruniversitäre Forschungseinrichtungen und 4 Industriepartner [16]. Klinisch, epidemiologisch und systemmedizinisch Forschende der Medizininformatik sowie Fachpersonal der Informationstechnologie arbeiten eng zusammen.

#### Konzept zur Kompetenzentwicklung.

Das in SMITH aufgebaute „SMITH Joint Expertise Center for Teaching“ (SMITH-JET) etablierte Angebote zur Aus‑, Fort- und Weiterbildung in der Medizininformatik und führte Fachwissen und Erfahrungen der Konsortialpartner aus Forschung, Industrie sowie den DIZ zusammen: Das Konsortium etablierte 3 neue medizininformatische Masterstudiengänge an den universitären Standorten Aachen, Jena und Leipzig. SMITH-JET initiierte die Weiterentwicklung medizinischer Anwendungs‑, Schwerpunkt- und Vertiefungsfächer in informatischen Studiengängen. Standortspezifisch wurde das Angebot vertiefender Lehrveranstaltungen für die Vermittlung digitaler Kompetenzen im Medizinstudium erweitert. SMITH-JET implementierte das SMITH-Shared Repository, welches online Lehr- und Lernmaterial für berufsbegleitende Fortbildung in SMITH verfügbar macht, und entwickelte einen Online-Kurs für Ärzt*innen und klinische Forscher*innen (Clinician Scientists) als Angebot der „SMITH-Academy“. Der Kurs befähigt sie dazu, die in den DIZ bereitgestellten Daten zur Beantwortung ihrer Forschungsfragen auszuwerten. Außerdem wurden befristet Rotationsstellen eingerichtet und ausgeschrieben, um klinisch Tätige für Datennutzungsprojekte und DIZ oder IT-Kräfte für klinische Studienprojekte freistellen zu können.

SMITH-JET entwickelte in Kooperation mit den anderen MII-Konsortien sowie der Gesellschaft für Medizinische Informatik, Biometrie und Epidemiologie (GMDS e. V.) den „Biomedical and Health Informatics Learning Objective Catalogue“ – kurz BMHI-Lernzielkatalog [17] – für die Lehre im Fach Medizininformatik. Dieser und weitere Lernzielkataloge können über die von SMITH-JET entwickelte Plattform HI-LONa (Health Informatics Learning Objective Navigator) eingesehen und kooperativ weiterentwickelt werden [18, 19].

#### Erfahrungen.

Die neuen Masterstudiengänge konnten aufgrund der Abstimmung zwischen medizinischer und mathematisch-naturwissenschaftlicher Fakultät zügig etabliert werden. Da diese Studiengänge offen sind für Teilnehmende mit heterogenem fachlichen Hintergrund, ergeben sich besondere didaktische Herausforderungen (z. B. fachsprachliche Kommunikationshürden, Unterschiede in Vorwissen, Methodenkompetenz und Lernverhalten). Sie wurden u. a. curricular durch profilspezifische Module, durch bedarfsbezogene Online-Angebote sowie durch kollaborations- und kommunikationsintensive Lernformate adressiert. Geeignete Maßnahmen müssen weiterentwickelt und erforscht werden.

In den DIZ werden Wissen und Kompetenzen benötigt, die so in den medizininformatischen Studiengängen noch nicht vermittelt werden. Der BMHI-Lernzielkatalog wurde um entsprechende Lernziele ergänzt. Auch in Zukunft sollten die Module in den Studiengängen und die Angebote für Fort- und Weiterbildung für neue DIZ-Mitarbeitende stetig angepasst werden.

#### Ausblick.

Alle entwickelten Qualifizierungsmaßnahmen sind nur im Verbund umsetzbar, der z. B. im oben genannten Aachener Studiengang durch gezielte Lehrimporte aus anderen SMITH-Standorten gelebt wurde. Die Partner werden weiterhin zusammenarbeiten, um den Digitalisierungsprozess im Gesundheitswesen durch ein lebendiges MII-weites Netzwerk in Deutschland und darüber hinaus zu gestalten.

Für dieses Netzwerk werden durch das Projekt baseTraCE sowohl die mit SMITH-JET entwickelten Lernzielkataloge und das Tool HI-LONa als auch die erarbeiteten Lehr‑/Lernmaterialien für andere Konsortien bereitgestellt. Parallel wird die SMITH-Academy zur konsortienübergreifenden MII-Academy ausgebaut.

### Vernetzung der MII-Nachwuchsgruppen

Innerhalb der MII werden aktuell 21 Nachwuchsgruppen (NWG) an den Universitäten gefördert, die neue Medizininformatik-Professuren eingerichtet haben.

#### Ausgangslage und Erfahrungen.

Da der Erfahrungsaustausch gerade für den wissenschaftlichen Nachwuchs wertvoll ist, wurde aus MIRACUM heraus der Austausch der NWG angestoßen. Ein erstes Treffen der NWG von MIRACUM und DIFUTURE fand beim MIRACUM-Symposium 2022 statt. Neben dem Aufbau einer Arbeitsplattform und einem weiteren Treffen 2023 fand im Rahmen der GMDS-Jahrestagung 2023 ein Barcamp mit dem Ziel statt, weitere NWG, auch außerhalb der MII, für das Netzwerk zu gewinnen.

#### Ausblick.

Im September 2023 startete das Vernetzungsprojekt „MII-Nachwuchsgruppen“^4^. Ziel ist es, den Austausch der NWG untereinander zu fördern und diese stärker in die MII und deren Gremien einzubinden. Zunächst wurden Bedarfe und Bedürfnisse der NWG sowie bereits bestehende (Vernetzungs‑)Aktivitäten ermittelt. In einem anschließenden Kick-off in Dresden wurde ein detaillierter Umsetzungsplan erarbeitet. Um die Sichtbarkeit der NWG zu erhöhen und deren Heterogenität zu unterstreichen, sind Öffentlichkeitsaktivitäten, z. B. gemeinsame Veröffentlichungen oder die Vorstellung der NWG über Steckbriefe, geplant. Thematische Synergien sollen für Kooperationen (z. B. gemeinsame Projekte und Publikationen) genutzt werden. Auch ein Austausch der NWG mit der Nachwuchsförderung der GMDS wird angestrebt. Über gezielte Veranstaltungen, wie Vortragsreihen, geknüpft an die Symposien der Konsortien, Saisonschulen oder Doktorand*innen‑/Habilitand*innentage, sollen zusätzlich der Austausch und die Sichtbarkeit der NWG innerhalb der MII unterstützt werden.

### Strategische Arbeitsgruppe Aus‑, Fort- und Weiterbildung (AFWB)

Für den nachhaltigen Erfolg der Maßnahmen in der akademischen Aus‑, Fort- und Weiterbildung ist eine konsortiumübergreifende Koordinierung der Aktivitäten und Angebote wichtig. So können übergreifende Schulungsbedarfe ermittelt sowie bereits lokal bestehende Angebote über die gesamte MII und darüber hinaus, z. B. für bestehende Clinician-Scientist-Programme, ausgeweitet werden. Diese Aufgaben werden von der strategischen Arbeitsgruppe „Aus‑, Fort- und Weiterbildung“ (AFWB) übernommen, in der alle Konsortien der MII vertreten sind und zusätzlich durch externe Expert*innen beraten werden. Die Koordination erfolgt durch den MFT.

#### Erfahrungen und bisherige Ergebnisse.

Die AFWB zeichnet sich durch eine enge Vernetzung mit Akteur*innen in und außerhalb der MII aus. Durch den MFT besteht eine unmittelbare Verbindung zum Nationalen Lernzielkatalog für Medizin (NKLM). Ebenso ist die AFWB eng mit der Fachgesellschaft GMDS verbunden. Bisherige Maßnahmen und Ergebnisse von MII und AFWB in Lehre und Weiterbildung wurden sowohl auf der Website der MII^5^ als auch in einem gemeinsamen Fachartikel dargestellt [2].

#### Ausblick.

In der MII hat sich eine übergreifende, nationale Koordination bewährt, in der die unterschiedlichen Ideen, Interessen und Ansätze ausgetauscht und gebündelt werden. So kann eine Brücke zu weiteren kompetenzbildenden Aktivitäten in anderen Netzwerken, wie zum Beispiel dem Netzwerk Universitätsmedizin (NUM), gebaut werden.

In der Ausbau- und Erweiterungsphase der MII agiert die AFWB als Initiator und Koordinator von Lernangeboten. Die technische Zusammenführung und das Angebot geteilter Schulungsressourcen erfolgen über die Weiterbildungsplattform baseTraCE^6^. Bereits identifizierte zusätzliche Weiterbildungsbedarfe im Bereich IT-Sicherheit und Einwilligungsmanagement im Regelbetrieb der Universitätsklinika sollen durch entsprechende Angebote gedeckt werden.

## Diskussion der Ergebnisse

Im Kontext der MII sind 48 neue Professuren an Universitäten und 3 an Hochschulen für angewandte Wissenschaften eingerichtet worden. Vernetzung hat sowohl innerhalb der Konsortien als auch in der Fachgesellschaft GMDS stattgefunden. Zur Kompetenzentwicklung sind 10 neue Studienangebote geschaffen worden: 1 Bachelor-, 6 konsekutive und 3 berufsbegleitende Masterstudiengänge (Tab. 1). Darüber hinaus wurden Lern- und Fortbildungsangebote etabliert, die standortübergreifend genutzt werden können, sowie Zertifizierungs- und Anerkennungsmöglichkeiten geschaffen.

**Tab. 1:** Neue Studienangebote in medizinischer Informatik und Datenwissenschaften, die im Kontext der MII geschaffen wurden

Hochschule	Abschluss	Bezeichnung	Kommentar
Universität Leipzig	M. Sc.	Medizininformatik	Konsekutiv
Universität Jena	M. Sc.	eHealth & Communication	Berufsbegleitend
RWTH Aachen	M. Sc.	Medical Data Science	Berufsbegleitend
Hochschule Mannheim	M. Sc.	Biomedizinische Informatik und Data Science (BIDS)	Berufsbegleitend, Kooperation mit MIRACUM, DIFUTURE und Graduate School Rhein-Neckar gGmbH
Universität Augsburg	B. Sc. M. Sc.	Medical Information Sciences	Konsekutiv
Medizinische Hochschule Hannover	M. Sc.	Biomedizinische Datenwissenschaft	Konsekutiv
TU München	M. Sc.	Data Engineering and Analytics	Konsekutiv
TU München	M. Sc.	Mathematics in Data Science	Konsekutiv
Universität Tübingen	M. Sc.	Medical Informatics	Konsekutiv

Die Aktivitäten richten sich an unterschiedliche Zielgruppen. Dazu gehören Personen mit fachlichem Hintergrund in Informatik, Medizin, Pflege, Bioinformatik, Biologie, Natur- und Datenwissenschaften. Für die MII erweisen sich Zusatzqualifikationen für Ärzt*innen in Informatik und für Informatiker*innen in Medizin als besonders wichtig. Sie können zu einer höheren Qualität bei der Softwareentwicklung und einer besseren Unterstützung der Behandlungsprozesse durch informationsverarbeitende Werkzeuge führen. In allen Konsortien haben digitale Lehr‑/Lernmethoden eine wichtige Rolle gespielt. Sie bieten Flexibilität, um standortübergreifend, interprofessionell und an individuellen Bedürfnissen orientiert auszubilden.

Mehrere der neuen Masterstudiengänge sind sowohl für Absolvent*innen aus Medizin, Gesundheits- und Naturwissenschaften als auch Informatik geöffnet. Dadurch werden die interprofessionelle Zusammenarbeit und Kommunikation gefördert. Die unterschiedlichen fachlichen Kompetenzen müssen durch spezielle didaktische Maßnahmen berücksichtigt werden, z. B. zu Beginn der Studiengänge durch verschiedene ergänzende Lehrangebote. Im Studienverlauf fördern kollaborationsintensive Lernformate die multiprofessionelle Integration.

Neben den Berufsgruppen sollten zunehmend auch Patient*innen digitale Kompetenzen aufweisen, um aktiv an der eigenen Gesundheit mitzuwirken und die Patientenperspektive in die medizinische Forschung einzubringen. Dafür sind eigene Ausbildungsformate notwendig und Öffentlichkeitsarbeit muss gestärkt werden.

Mit dem Aufbau der DIZ und der übergreifenden Infrastrukturen zum Data Sharing hat sich die begleitende Aus‑, Fort- und Weiterbildung als ein Erfolgsfaktor in der MII etabliert. Durch die zunehmende Verschmelzung der Konsortien sowie den zukünftigen Zusammenschluss mit dem NUM konvergieren auch die bestehenden Lehr‑/Lernformate und -inhalte. Dies wird durch die strategische Arbeitsgruppe AFWB koordiniert. Ziel ist es, abgestimmte Materialien herauszugeben, die sich an den erarbeiteten Lernzielen orientieren. Erfahrene Stärken und Schwierigkeiten videobasierter Lernangebote förderten die Nutzung didaktisch angemessener Formate (z. B. Microlearnings, E‑tivities, Podcasts). Saisonschulen erwiesen sich mehrfach als förderlich.

Die zunehmende Verschmelzung der Konsortien zeigt sich auch in dem Übergang der SMITH-Academy in die MII-Academy. Damit wird die Zielgruppe um Clinician Scientists als Datennutzende erweitert. Insbesondere die aufgebauten Services (z. B. Machbarkeitsanfragen, Antragsportal, Datenschutz und Ethik, Infrastrukturen zur verteilten Analyse) und ihre Nutzung sollen bekannt gemacht werden.

Gelebte Kooperation ist ein entscheidender Erfolgsfaktor der Kompetenzentwicklung in der MII. In der Aufbau- und Entwicklungsphase der MII wurde bereits mit dem NUM kooperiert. Dies soll ausgeweitet werden. Es werden sowohl Datennutzungsprojekte ausgeschrieben als auch gemeinsame Schulungen im Bereich IT-Sicherheit und Consent-Management angeboten.

Im *NKLM* Version 2.0 wurden die Lernziele für das Medizinstudium und das Zahnmedizinstudium konsentiert und veröffentlicht. Dazu gehört auch der Katalog „Digitale Kompetenzen“, der in Zusammenarbeit mit der GMDS entwickelt wurde und Lernziele aus der medizinischen Informatik [20] mit den medizinischen Lernzielen verknüpft. Die Studierenden sollen notwendige Kompetenzen im Umgang mit medizinischer Informationstechnologie erwerben und in ihrer ärztlichen Tätigkeit anwenden können.

Vertreter*innen von MIRACUM und DIFUTURE haben *Auslandsreisen *unternommen, um sich über aktuelle Entwicklungen zu informieren. Es wurde festgestellt, dass die Angebote für Studierende der Medizin und der medizinischen Informatik in den USA insgesamt vergleichbar mit den eigenen Angeboten sind. Ein wichtiger Unterschied besteht bei PhD-Programmen, die in den USA oft stärker strukturiert sind und in relativ kurzer Zeit abgeschlossen werden können. Die Entwicklung strukturierter Promotionsprogramme in medizinischen Datenwissenschaften in Deutschland könnte von den Lernangeboten profitieren, die aus der MII entstanden sind.

### Ausblick

Damit die Angebote zur Qualifizierung von Personal in der MII standortübergreifend genutzt werden können, werden sie konsolidiert und auf die in den Lernzielkatalogen formulierten Kompetenzen abgebildet. Sie werden systematisch erfasst und mit Metadaten so beschrieben, dass sie anhand der angestrebten Kompetenzen gut auffindbar sind und den FAIR-Kriterien^7^ genügen [21]. Eine zentrale Rolle wird hierbei die gemeinsame Koordinierung der Lernangebote und Kompetenzprofilbildung spielen, die mithilfe von baseTraCE einen Beitrag zur Harmonisierung leisten soll.

Die zukünftigen Aktivitäten sollen ein besonderes Augenmerk darauf legen, dass digitale Kompetenzen auch für Datennutzende notwendig sind. Nur dann kann datengetriebene Medizin zu einer Verbesserung von Forschung und Versorgung führen. Deshalb beschreibt die MII-Academy die aufgebauten Data-Sharing-Services und -Prozesse. Dazu zählen sowohl die DIZ als auch die Plattformen (z. B. zur Datenbeantragung und verteilten Datenanalyse), die eine übergreifende Nutzung medizinischer Daten erlauben. Zielgruppe sind Clinician Scientists und die in der medizinischen Forschung Tätigen. Alle Konsortien haben sich darauf verständigt, sie in den Fakultäten gemeinsam zu bewerben.

Auch in der Ausbildung von Studierenden werden neue Wege beschritten. So sollen im Modellstudiengang Humanmedizin „MEDiC“ der Technischen Universität Dresden und des Klinikums Chemnitz die Möglichkeiten von Digital Health integraler Bestandteil der praxisnahen Ausbildung sein. Dabei können die MII-Angebote genutzt werden, um ein gemeinsames Verständnis von Themen wie Digital Health, E‑Health, Big Data, künstliche Intelligenz etc. zu entwickeln. Die Kompetenzvermittlung beschränkt sich nicht auf die Studierenden, sondern auch die Lehrenden und Mentor*innen sollen weitergebildet werden. Die Saisonschulen oder digitalen Lehrangebote der MII können hierfür genutzt werden. Diese Grundlage kann in Zukunft ebenfalls genutzt werden, um ärztliches, pflegerisches und weiteres medizinisches Personal in nicht universitären Krankenhäusern so fortzubilden, dass digitale Kompetenzen für die tägliche Arbeit aufgebaut werden. Wenn sich die Zielgruppe der aus der MII entwickelten Lernangebote dadurch erheblich vergrößert, sind weitergehende Ressourcen notwendig, um das auch langfristig anbieten zu können.

## Fazit

Der Erfolg der MII hängt wesentlich von der Akzeptanz der Gesellschaft für die Mehrfachnutzung medizinischer Daten sowohl in der Versorgung als auch in der Forschung ab. Dafür erforderliche Information wird der Bevölkerung durch Öffentlichkeitsarbeit der MII bereitgestellt. Darüber hinaus besteht in der Gesellschaft ein enormer Bedarf an Kompetenz im Umgang mit medizinischen Daten und digitalen Werkzeugen in der Medizin („digital/medical literacy“). Kompetenzentwicklung ist daher auch in den Schulen und in der Erwachsenenbildung erforderlich.

## Footnotes

1 https://difuture.de/strengthening-medical-informatics/ (letzter Zugriff 04.04.24).

2 https://digitalisierungdermedizin.de/.

3 https://cutt.ly/JXsNCM3.

4 https://www.medizininformatik-initiative.de/de/vernetzung-der-nachwuchsforschungsgruppen-der-medizininformatik-initiative.

5 https://www.medizininformatik-initiative.de/de/konsortien/medizininformatik-lehre-und-fortbildung (letzter Zugriff 04.04.24).

6 https://www.gesundheitsforschung-bmbf.de/de/basetrace-medizininformatik-struktur-basisdienstleistungen-fur-die-aus-und-weiterbildung-16899.php (letzter Zugriff 04.04.24).

7 FAIR-Kriterien: Daten sollen auffindbar („findable“), zugänglich („accessible“), interoperabel („interoperable“) und wiederverwendbar („reusable“) sein.
